# The Global Food System as a Transport Pathway for Hazardous Chemicals: The Missing Link between Emissions and Exposure

**DOI:** 10.1289/EHP168

**Published:** 2016-07-06

**Authors:** Carla A. Ng, Natalie von Goetz

**Affiliations:** 1 Department of Civil and Environmental Engineering, University of Pittsburgh, Pittsburgh, Pennsylvania, USA; 2 Institute for Chemical and Bioengineering, ETH Zurich (Eidgenössische Technische Hochschule Zürich), Zurich, Switzerland

## Abstract

**Background:**

Food is a major pathway for human exposure to hazardous chemicals. The modern food system is becoming increasingly complex and globalized, but models for food-borne exposure typically assume locally derived diets or use concentrations directly measured in foods without accounting for food origin. Such approaches may not reflect actual chemical intakes because concentrations depend on food origin, and representative analysis is seldom available. Processing, packaging, storage, and transportation also impart different chemicals to food and are not yet adequately addressed. Thus, the link between environmental emissions and realistic human exposure is effectively broken.

**Objectives:**

We discuss the need for a fully integrated treatment of the modern industrialized food system, and we propose strategies for using existing models and relevant supporting data sources to track chemicals during production, processing, packaging, storage, and transport.

**Discussion:**

Fate and bioaccumulation models describe how chemicals distribute in the environment and accumulate through local food webs. Human exposure models can use concentrations in food to determine body burdens based on individual or population characteristics. New models now include the impacts of processing and packaging but are far from comprehensive. We propose to close the gap between emissions and exposure by utilizing a wider variety of models and data sources, including global food trade data, processing, and packaging models.

**Conclusions:**

A comprehensive approach that takes into account the complexity of the modern global food system is essential to enable better prediction of human exposure to chemicals in food, sound risk assessments, and more focused risk abatement strategies.

**Citation:**

Ng CA, von Goetz N. 2017. The global food system as a transport pathway for hazardous chemicals: the missing link between emissions and exposure. Environ Health Perspect 125:1–7; http://dx.doi.org/10.1289/EHP168

## Introduction

Food is a major pathway for human exposure to potentially hazardous chemicals [National Research Council (NRC) 2012] and may contain a wide variety of chemicals that enter at many points along the value chain. Chemicals are used to increase efficiency and yield during production (pesticides, hormones, antibiotics), may be applied to increase stability (surface treatments, preservatives, packaging ingredients) and compatibility (emulsifiers) during processing, or may inadvertently end up in food because they are present in the environment, particularly if they are persistent under environmental conditions. Thus, even chemicals that have been largely banned, such as dichlorodiphenyltrichloroethane (DDT) and polychlorinated biphenyls (PCBs), continue to be regularly detected in foods (Schecter et al. 2010). Chemicals that are intentionally applied to food are relatively strictly regulated in most countries [e.g., by maximum residue levels (MRLs) set by national agencies or via the international Codex Alimentarius (FDA 2016; Health Canada 2010; Veggeland and Borgen 2005)]. MRLs have also been set for a small number of environmental pollutants (e.g., dioxins), but these substances are typically only detected via “spot checks” that are limited in the number of target chemicals and the number of food samples they can cover (European Parliament 2006; Malisch and Kotz 2014). As the complexity of our food system grows, it becomes increasingly difficult to consistently monitor the presence of contaminants in food. As set out in the landmark report, *Exposure Science in the 21st Century*, exposure science will play a critical role in supporting policies that ensure the safety and sustainability of the food supply (NRC 2012). However, in order to use exposure data to craft control and regulatory measures, exposures must be adequately linked to the sources of the chemical(s) in question, and in our current food system, sources can be far removed (both geographically and via many processing steps) from the site of exposure.

Our food system is increasingly globalized. Food trade increased in value from 438 billion USD in 1998 to 1.06 trillion USD in 2008 (Ercsey-Ravasz et al. 2012), growing faster than production itself. At the same time, trade has shifted from fresh foods and agricultural raw materials to more complex, processed food products (Ercsey-Ravasz et al. 2012); processed food now accounts for > 50% of total food exports globally (Jongwanich 2009). Thus, tracing chemical residues in foods back to their sources depends not only on their terroir—a concept often used to connote a set of flavor characteristics imparted to foods by their local growing conditions, but which is also critical to determining a food’s environmental contaminant profile—but also on the totality of chemical transfer during production, processing, packaging, and storage. At each of these steps, the number, identity, and concentration of chemicals may also be influenced by environmental and regulatory differences between countries and regions.

One approach to understanding chemical fate in foods and subsequent human exposure is through the use of models. Two types of models are typically developed for this purpose: bioaccumulation models and human exposure models. Bioaccumulation models attempt to trace chemical accumulation from the environment through the food web into different foods (Streit 1992). Although sophisticated models exist that combine the global distribution of chemicals (as driven by processes in the air, water, and soil) with key predator–prey interactions (Breivik et al. 2010; Czub and McLachlan 2004; Rosenbaum et al. 2011; Undeman et al. 2010), such models do not consider the many intermediate steps food often takes between harvest and consumption. Human exposure models, in contrast, often rely on measured levels in the foods of interest, which are combined with consumer data on consumption, body weight, age, and sex. Human exposure is then calculated for a specific chemical by multiplying the chemical’s concentration in a food item with the consumption of that food item, then adding up the exposures from all single food items to an aggregate exposure (Trudel et al. 2011; von Goetz et al. 2010). At present, the most sophisticated models for human exposure to chemicals via food also take into account the packaging (Oldring et al. 2014), but the origin of the food is often difficult to assess. Although a few countries have instituted mandatory country-of-origin labeling, it usually only applies to specific sectors (e.g., seafood) (Joseph et al. 2014), and labeling practices may change as a result of political or trade pressures [Agricultural Marketing Service (AMS) 2016; Newman et al. 2014; Tracy 2015]. Therefore, food origin is typically not addressed within human exposure models. Because bioaccumulation models focus on chemical transfer from the source to the surrounding environment and local organisms without incorporating human-mediated transport (i.e., food trade), and human exposure models assess the uptake of chemicals from food without explicitly accounting for the food origin, the link between the chemical source and human exposure is effectively broken. What is missing between the two approaches is an explicit consideration of the industrial food web through which the majority of people now obtain their food.

### Objectives

In this work, we argue that a fully integrated approach is needed to investigate how the distribution of chemicals in the environment influences the exposure of consumers within the context of the modern food system. Only by explicitly accounting for the sources of different chemicals in foods can we predict human exposure to the myriad of health-relevant chemicals they contain, despite limited analytical resources, and conduct sound risk assessments and effective risk abatement strategies. Here, we present a conceptual framework to further the science of human exposure to chemicals via one of the most important, and complex, exposure pathways—the global industrial food web.

## Discussion

### Chemical Transfer to Food During Production

The distribution of chemicals in the environment depends on their emissions, physicochemical properties, and environmental transport processes. Direct (point-source) and diffuse emissions can occur throughout a chemical product’s life cycle, from production through use and disposal. Thus, chemical emissions into environmental compartments may be determined by levels of industrial activity (e.g., solvent releases into rivers), levels of agricultural activity (e.g., herbicide use on soil), climatic patterns that influence disease vectors (e.g., global insecticide use), regulations (e.g., aerial vs. ground application of pesticides), or population density (e.g., personal care product releases into sewage systems: shower gel and shampoo from showering, shaving gel from shaving). Once released, the chemical properties themselves—in particular, volatility, partition coefficients, and degradation rates—are key. Finally, mechanisms of transport, such as advection with wind or ocean currents, shape the way contaminants move on a global scale.

During crop production or growth of livestock, intentionally applied chemicals include pesticides, growth stimulants, and therapeutic drugs. The residue level of specific pesticides depends on their use and therefore on both the crop and the regional pests; thus, the “terroir” of the food is, to some extent, a predictor of the residue level of a specific pesticide and the human exposure to residues in consumed foods. For example, because of climatic conditions, insecticide use in Spain is much more common than in Germany or Switzerland, where more herbicides are used (EuroStat 2016).

At the same time, environmental contaminants—in particular, persistent pollutants—may enter foods by transfer from air, water, or soil. Some persistent organic pollutants (POPs) such as DDT and its toxic metabolites (collectively, ΣDDT), are distributed according to agricultural or vector control activity. DDT was banned from agricultural use in most industrialized countries in the 1970s and 1980s (Rogan and Chen 2005). But owing to its persistence, ΣDDT is now globally distributed, with hotspots in regions where DDT is still used for malaria vector control (Leslie et al. 2013). ΣDDT is transferred from air to water and soil, readily accumulates in lipophilic materials, and biomagnifies in food webs, leading to high concentrations in foods such as butter and fatty fish. Thus, whereas concentrations in butter depend largely on transfer of ΣDDT from air to grass and subsequent consumption by dairy cows (Kalantzi et al. 2001; McLachlan 1994), for fish, the concentration depends not only on the region where they are caught but also on the species and its position in the food chain (McIntyre and Beauchamp 2007). POPs generated within the technosphere, such as PCBs, may enter the environment through different pathways. Although PCBs were banned in the 1970s, they continue to be released from electrical transformers and building materials produced before the ban (Kohler et al. 2005). This continued release gives rise to regional hotspots throughout the world (He et al. 2015; Weber et al. 2011). Despite these differences, the global distribution of many volatile and semi-volatile POPs, including ΣDDT and PCBs, occurs mainly via atmospheric transport (Lohmann et al. 2007), with an additional component driven by ocean currents. Because of the movement of these currents, longitudinal dispersion of chemicals is generally faster than latitudinal transport. Transport across the equator, for both air and water currents, is particularly slow. Therefore, global-scale chemical fate models typically assume relatively rapid distribution of chemicals within latitudinal bands and much slower transport across the equator (Scheringer 2009).

Several multimedia bioaccumulation models have been developed to link chemical emissions and environmental distribution with accumulation in human food chains. ACC-HUMAN includes both aquatic and agricultural food chains, but it lacks spatial resolution (Czub and McLachlan 2004). Other authors subsequently extended the model by linking it to a more complex fate model (CoZMo-POP2) to consider the effects of non–steady-state emissions patterns (Breivik et al. 2010) or the influence of climatic regions (Undeman et al. 2010) on bioaccumulation. However, in both cases it was assumed that concentrations in the diet came from the local environment of the exposed population.

The USEtox model (Rosenbaum et al. 2011) for assessing human exposure to toxic chemicals within life-cycle assessment calculates the transfer of chemicals from the production environment into meat and milk, thus theoretically accounting for the chemical concentration at the site of food production without assuming that humans are directly exposed to the same environment (so-called “production-based intake scenarios”). However, food trade flows are not explicitly included in this model, and the description of the environment has no spatial resolution (Henderson et al. 2011).

One of the best examples of combining a spatially explicit chemical fate model with food production and consumption data is the study by MacLeod et al. (2004), which coupled the Berkeley–Trent (BETR) North America contaminant fate model with regional food production and consumption data to estimate the exposure of the North American population to a suite of air contaminants. The authors showed that a spatially explicit approach is essential for chemicals for which the ingestion pathway is dominant (that is, for which the chemicals accumulate from the air into food and are subsequently ingested) and for those chemicals with relatively low environmental mobility, where the proximity of the site of food production to the source of the chemical becomes more important (such as benzo[*a*]pyrene). However, that study assumed that all foods were produced in North America, and the authors did not account for any regional variation in the foods consumed.

Therefore, models are already in place that can address spatially explicit emissions and chemical fate and bioaccumulation. However, these models fail to account for the transport of chemicals via food trade, which may follow pathways that differ from the distribution of chemicals in the environment via natural processes like advection with air and water. Additional data or methods of parameterization will be needed to adequately link these models to spatially resolved descriptions of consumption.

### Chemical Transfer to Food via Storage, Processing, and Packaging

Once a chemical has gone through the processes of emission, environmental distribution, and accumulation in a given food matrix, it enters, together with the food, another complex set of processing steps embodied in the industrial food system, which, being global, may occur in different places. Over the past 30 years, there has been a marked shift in traded commodities away from fresh foods and basic agricultural staples towards more meat, processed foods, and high-quality, off-season, or exotic foods (Ercsey-Ravasz et al. 2012; Hazell and Wood 2008; Jongwanich 2009). Global food trade has more than doubled in the last three decades, supported in large part by increasing wealth, with rises in trade relationships and trade value following increasing GDP, and outpacing both global population and global crop yield (Dalin et al. 2012; D’Odorico et al. 2014).

With this globalization and industrialization, food chains are becoming longer and more complex. Some supply chains, such as those for fresh fish, now undergo different processing steps in different countries (Schröder 2007). For processed foods, cross-contamination can occur at any step, and production origin data alone are not sufficient (Kruse 1999). Given this complexity, it is extremely difficult to determine the origin of particular foods (LeBlanc et al. 2015).

Unlike environmental chemicals that can be traced back to the origin of foods, the use of food additives (a broad category that includes nutritional additives, processing agents, preservatives, and sensory agents) can not only vary according to the region where processing takes place (owing to regional legislation, culture, or know-how) but also depend on product type and company procedures. Some toxicologically relevant compounds, such as polycyclic aromatic hydrocarbons (PAHs), are imparted to foods during processing methods such as smoking or adding smoke flavor (Gomaa et al. 1993). Such chemical transfer can depend on the specific procedures used but is also dependent on packaging and shelf life. For example, acrylamide levels in coffee are lower when vacuum roasting is used than when conventional roasting methods are employed (Anese et al. 2014), and the levels decrease over time in roast coffee products stored at ambient temperatures (Lantz et al. 2006). Packaging itself can release substances such as fluorinated compounds or plasticizers (Bhunia et al. 2013). These substances can also be introduced by specific processing steps such as the handling of meat with PVC gloves (Tsumura et al. 2003) or the use of plastic tubing for milk (Ruuska et al. 1987).

Thus, for effective modeling of chemical fate in food, the processing, packaging, and storage of foods need to be considered. A number of models are available for the optimization of food processing or storage; examples include models for the melting and crystallization of fats (Himawan et al. 2006), spray drying to convert liquids into powders (Keshani et al. 2015), and the development of suitable packaging sizes or materials (Sousa Gallagher et al. 2011). Exposure models that take into account processing-induced changes to chemicals in food are scarcer. For pesticides, where the influence of processing on pesticide concentrations must be evaluated as part of the registration procedure [European Parliament (EP) 2009], a fate model was proposed for pesticides applied to potatoes; this model includes a fixed processing factor and the effects of storage in the calculation of daily intake (Juraske et al. 2011). The probabilistic MCRA model (van der Voet et al. 2015) can account for uncertainty by using a range of processing factors, but data that can be used to estimate processing factors are very scarce (van Ooijen et al. 2009). In addition, processing factors are specific to a single substance of interest and do not account for other substances formed during processing.

The FACET model has been recently developed for chemicals that migrate from packaging materials (Oldring et al. 2014); this model calculates migration into specific foods based on classical migration studies of food simulants or on the composition of the food contact material. The U.S. Food and Drug Administration (FDA) employs a similar approach based on food-specific migration and packaging factors (FDA 2007). However, even if these approaches work well for packaging, they remain somewhat isolated because only migration of chemicals from packaging material is considered: Moerman and Partington (2014) showed that often, the same chemicals are also released from processing containers (Moerman and Partington 2014) and therefore add to the concentration in a packaged food.

Hence, for all steps in the food system (production, processing, storage, and packaging), efforts are underway to understand which chemicals can contaminate food and under what circumstances, but the models are not comprehensive. For some chemicals, the integration of all steps is not necessary (e.g., when the chemicals enter only at the very end of the value chain, i.e., mostly the packaging), but most environmental and some processing chemicals are modified or enter at the beginning or in several parts of the food system. For those chemicals, only an integrated assessment can deliver suitable information for designing the most effective intervention strategies or for extrapolation of analytical data.

### Food-Borne Human Exposure to Chemicals: From Local to Global Diet Modeling

The origins of many foods have changed in recent years, with developing countries exploiting new markets in Europe and North America (e.g., for wine and fish) and increased trade taking place between developing countries [e.g., between China and Brazil (Dalin et al. 2012; Hazell and Wood 2008)]. At the same time, food markets have become more integrated and global and are now dominated by a few large international trading companies (Hazell and Wood 2008).

Current exposure assessments for food-borne chemicals mostly rely on the combination of chemical concentrations in food with data on the consumption of foods by a certain population [European Food Safety Authority (EFSA) 2013, 2015]. Concentrations for a limited number of chemicals are available from the open literature and dedicated surveys. It is assumed that the food items acquired for analysis in a specific region/country, which is typically indicated in the respective analytical study, is also consumed in that region/country (EFSA 2015); that is to say, it is assumed that a concentration measured in fish from Ireland determines the exposure of Irish people. This assumption is mostly valid, but the food basket analyzed needs to be representative for the studied population, requiring large data sets specific to a region. Because substance concentrations vary among regions owing to environmental factors [e.g., concentrations of polybrominated diphenyl ethers in Irish fish differ according to where they are caught (Trudel et al. 2011)] or specific processing, they often cannot be extrapolated from one region/country to another. Consequently, data gaps or inconsistent data sets are very common. Nevertheless, because of global trade, the same food may be consumed in different regions. Knowing which components of a regional diet are produced locally (e.g., fresh tomatoes) and which are traded and thus common in other regions (e.g., canned tomatoes) could help to fill those data gaps, but correct identification of the food items that can be extrapolated to a different region would require the origin of the food to be labeled.

Some important new developments are occurring with consumption surveys. Although it is a difficult task, food packaging has been included in the description of foods in some recent European consumption surveys (Merten et al. 2011), and it will continue to be included (Schweter et al. 2015). If food origins were labeled, they could also be integrated in such a survey, allowing us to allocate exposure via food to all components of the food system, from field to fork.

### Putting it Together

**Figure 1:**
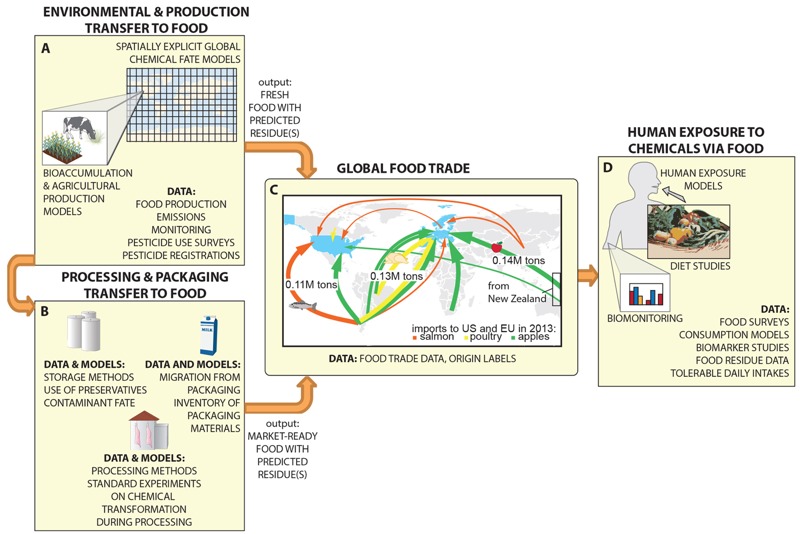
(*A*) Environmental and production-related chemical transfer to food. Several models are available for predicting global chemical distribution. Production-specific models are also available to assess transfer of pesticides and other production-related chemicals to meat, milk and produce. Examples of data that can be used to parameterize and validate these models are presented. (*B*) Storage, processing, and packaging-related chemical transfer to foods. Some initial models have been developed to begin to address these complex mechanisms. (*C*) Food trade within the global industrial food system. Here, we illustrate the highest import flows for salmon, poultry, and apples into the United States and Europe (defined as the EU-28 plus Switzerland and Norway) from the rest of the world in 2013. Line widths for food flows are proportional to net weight in metric tons [extracted from the Comtrade database (DESA/UNSD 2014)], and only flows > 25,000 metric tons are included for clarity. (*D*) Human exposure via food. Human exposure models can be coupled to a wide range of data types, including food surveys, food basket studies, and measured food residue data. Complementary data, such as data from human biomonitoring, can be used to evaluate model outputs. Conversely, a better understanding of the global food system embodied in panels (*A–C*) can help with tracing the origins of chemicals identified in biomonitoring or biomarker data and provide opportunities for eliminating key sources.

The elements needed for an integrative understanding of chemical transport in the global food system include both models and corresponding data (Figure 1). A number of models already exist for certain components of the system, and ancillary data are available to refine and expand the applicability domains of the models. For the transfer of chemicals to food during the production process (including environmental contaminants), robust spatially explicit chemical fate models are available (Figure 1A). For example, BETR Global expands the spatially explicit Berkeley–Trent model to a global scale (MacLeod et al. 2011). Such a model could be linked to a region-specific bioaccumulation or pesticide transfer model [e.g., McLachlan (1994), Juraske et al. (2011), or Fantke and Jolliet (2016)] to predict chemical concentrations in fresh foods. By using ancillary data such as emission inventories, national food production statistics and wildlife monitoring data, spatially resolved global-scale predictions of contaminant residues could be generated. These predictions could then serve as inputs to models of storage, processing, and packaging (Figure 1B).

Models describing food processing and packaging remain in the early stages of development. To our knowledge, at the present time, processing is only taken into account by the inclusion of specific processing factors. These factors need to be determined for each chemical separately under standardized conditions. Because processing factors are not available for many chemicals, it would be important to identify model chemicals and adequate model processing procedures, to test relevant combinations, and to use quantitative structure-activity relationships (QSARs) to extrapolate the results to other chemicals. Some standard model procedures (e.g., for cooking, baking, or smoking under different temperature and pH regimes) that can predict how chemicals in food are transformed during processing have been identified for pesticides (EP 2009), but it remains to be tested whether these are sufficient and can also apply to other chemicals present in food. Existing models such as FACET and MCRA (discussed in “Chemical Transfer to Food via Storage, Processing, and Packaging”) could also be used to predict the transfer of chemicals to processed and packaged foods (Oldring et al. 2014; van der Voet et al. 2015).

After this step, both fresh foods with residues resulting from production and environmental contamination, as well as processed foods containing additional chemicals transferred during storage, processing, or from packaging, need to be assigned to the appropriate, region-specific, human populations. However, a crucial data gap in many countries is the origin of foods. At the present time, only a few countries have instituted mandatory country-of-origin labeling regulations, which often apply to a limited selection of foods (Woolfe and Ditton 2013). Research on food safety and the spread of pathogens in the food system have illustrated the difficulty in tracing a particular item all the way from field to fork, but this is a critical need given the rapid global reach of contaminated foods, as highlighted by recent food scares (Tauxe et al. 2010). There are, however, some sources of data and model approaches that are already available. To help fill this gap, we propose that trade flow data, coupled with production and consumption data, can be used as a surrogate for country-of-origin information for foods (Figure 1C). Technically, the inclusion of trade flows might be possible using simple material flow analysis, which can be used to build national or global trade networks via an input–output approach to account for material flows using publicly available data on production, consumption, imports, and exports (Fischer-Kowalski et al. 2011).

To produce the panel in Figure 1C, we compiled data regarding the global imports of three types of fresh food—apples, chicken and salmon—to the United States and Europe from the rest of the world. We highlight in the figure the highest food flows by weight, based on 2013 trade data from the United Nation’s Comtrade database (DESA/UNSD 2014). The arrows illustrating food flows from each exporting country into the United States and Europe are scaled by weight, and for clarity, we only show flows > 25,000 metric tons (essentially the top 1–5 food flows in each category). The top flow for each food is labeled with the flow, for the year 2013, in million metric tons. Included in “Europe” is the EU-28 classification from Comtrade, plus Switzerland and Norway. The Comtrade database contains self-reported information from exporting and importing countries for a wide variety of fresh and processed foods. Although these data contain some uncertainty, they can serve as a starting point for developing mass flow models for the movement of food between countries. Such an approach has been successfully used to construct virtual water trade networks, models of the trade in water used in the production and transport of foods to places where the food is consumed (see, e.g., Dalin et al. 2012; Grote et al. 2008; Yang et al. 2006). Subsequent studies also investigated the flow of nutrients embodied in crops. Unlike water, nutrients (such as nitrogen and phosphorous) are not virtually traded but are actually transported within the food system (Lassaletta et al. 2014). An effective accounting of chemical transport via foods can benefit from the work on mapping virtual water and nutrient trade networks, but additional components, such as coupling with GIS, may be needed (LeBlanc et al. 2015; NRC 2012).

Of particular note in the trade flows pictured in Figure 1C is the prevalence of food transport across the equator. This transport results not only from flows of off-season fruits (e.g., apples from New Zealand to Europe) but also from flows of meat (e.g., chicken from Brazil to Europe) and fish (e.g., salmon from Chile to the United States). Unlike environmental flows of chemicals with air and water, which have relatively rapid transport within each hemisphere and slower transport across the equator (Scheringer 2009), flows of chemicals in traded food readily and rapidly cross the equator.

With these trade flows in place, the chemical accumulation and transfer models can be linked to human exposure models (Figure 1D). Here, additional data can be used not only to construct population-specific exposure models (e.g., using individual food surveys or regional food basket studies) but also to validate the integrated model outputs by comparing predicted food levels and exposures to food residue data, biomonitoring data, and biomarker studies. The types of data used will depend in large part on the contaminants of interest. Some contaminants are sufficiently specific or unique such that their presence alone in a food is sufficient to track origin and even time of exposure. For example, the nuclear accident in Chernobyl, Ukraine, serves as an early and excellent example of global human exposure to a highly region-specific contamination event (Anspaugh et al. 1988). More recently, the effects of the Fukushima, Japan, incident have been traced through the contamination of seafood and have even been used to help reconstruct the migration patterns of bluefin tuna (Madigan et al. 2014). Depending on the type of residue considered, human biomonitoring or biomarker data could directly inform chemical fate and bioaccumulation models (McKone et al. 2007; Shin et al. 2013). For contaminants having diverse sources in different regions, exposure data will represent an aggregate picture, and reconstruction of exposure pathways will be more complex.

## Conclusions

A more comprehensive approach is needed to understand how the food system influences the transport of chemicals on a global scale and what implications this transport has in terms of human exposure, environmental health, and food safety. To integrate many different models and data sources, as suggested here, the model scales will need to be matched. It will likely be necessary to refine the structure of the models considered, particularly for emissions, where models with sufficient spatial resolution to capture chemical hot spots may be required. Understanding and managing chemical transport through the global food system is a highly ambitious endeavor, but it relies largely on integrating existing research knowledge and infrastructure.

By explicitly including the role of the food system in the fate of environmental contaminants, chemical fate and exposure scientists will be able to address the following key research needs:

understanding the movement of contaminants in ways not currently predicted by global chemical fate models
achieving better understanding of human biomonitoring data and developing strategies to reduce exposure to contaminants in food
identifying food production regions and food items that may be vulnerable to certain types of contamination, providing the basis to reduce contaminant transfer by optimizing crop–region relationships (which crops are best grown where).

As shown in earlier studies of “biotransport” of contaminants by migrating salmon (Ewald et al. 1998; Hites et al. 2004; Krümmel et al. 2005), chemical inputs into a region via unorthodox sources (such as migrating wildlife or human transport) may be more important than inputs via air and water flows. With a more complete understanding of the food system, from production through processing, maximal exposures could be predicted with increased accuracy, and potentially hazardous processing steps could be identified and changed. In addition, environmental contaminants could be traced back to their point of origin, allowing generalization or extrapolation of specific analytical findings of chemicals in food, which ultimately aids in leveraging of small data sets. Simultaneously, interventions could be steered more effectively. Given the increasing pressures on our agricultural system and the need to feed a growing global population, the global food system will continue to expand, and it is unlikely that its complexity will decrease. However, by understanding how terroir influences the presence of environmental chemicals in food and how the complex chains of food transport, processing, and packaging contribute to the overall contaminant profile in market-ready foods, we can design a food system that minimizes exposure to potentially hazardous chemicals.
